# Challenges and strategies for sustaining youth-friendly health services — a qualitative study from the perspective of professionals at youth clinics in northern Sweden

**DOI:** 10.1186/s12978-016-0261-6

**Published:** 2016-12-21

**Authors:** Suzanne Thomée, Desiré Malm, Monica Christianson, Anna-Karin Hurtig, Maria Wiklund, Anna-Karin Waenerlund, Isabel Goicolea

**Affiliations:** 1 Unit of Epidemiology and Global Health, Department of Public Health and Clinical Medicine, Umeå University, Umeå, SE-90187 Sweden; 2 Department of Nursing, Umeå University, Umeå, Sweden; 3 Department of Community Medicine and Rehabilitation, Physiotherapy, Umeå University, Umeå, Sweden

**Keywords:** Youth-friendly health-care services, Youth clinics, Thematic analysis, Health professionals, Youth health

## Abstract

**Background:**

Youth-friendly health-care services — those that are accessible, acceptable, equitable, appropriate and effective for different youth subpopulations – are beneficial for youth health, but not easy to implement and sustain. Sweden is among the few countries where youth-friendly health-care services have been integrated within the public health system and sustained for a long time.

This study explores the challenges and strategies in providing sustainable youth-friendly health-care services, from the perspective of professionals working in youth clinics in northern Sweden.

**Methods:**

Eleven semi-structured interviews with various health-care professionals working in youth clinics in northern Sweden were conducted. The interviews were transcribed verbatim, and analysed using thematic analysis in relation to the World Health Organization domains of youth friendliness.

**Results:**

Four themes emerged from the analysis of the data: 1) ‘Meeting youths on their own terms – the key to ensuring a holistic and youth-centred care’ was related to the acceptability and appropriateness of the services; 2) ‘Organizational challenges and strategies in keeping professionals’ expertise on youth updated’ referred to the domain of effectiveness; 3) ‘Youth clinics are accessible for those who know and can reach them’ was related to the domains of accessibility and equity, and 4) ‘The challenge of combining strong directions and flexibility in diverse local realities’ focused on the struggle to sustain the youth clinics organization and their goals within the broader health system.

**Conclusions:**

Professionals working in youth clinics are perceived as motivated, interested and knowledgeable about youth, and the clinics ensure confidentiality and a youth-centred and holistic approach. Challenges remain, especially in terms of ensuring equitable access to different youth subpopulations, improving monitoring routines and ensuring training and competence for all professionals, independently of the location and characteristics of the clinic. Youth clinics are perceived as an indisputable part of the Swedish health system, but organizational challenges are also pointed out in terms of weak clear directives and leadership, heavy workload, local/regional diversity and unequitable distribution of resources.

**Electronic supplementary material:**

The online version of this article (doi:10.1186/s12978-016-0261-6) contains supplementary material, which is available to authorized users.

## Plain English summary

Health-care services can contribute to improving youth health, as long as they are accessible, acceptable, equitable, appropriate and effective for different youth subpopulations — what the World Health Organization calls ‘youth-friendly health-care services’. However, few countries can claim that they have a health-care system that is friendly for youths. This study was conducted in Sweden, which is amongst the few countries where youth-friendly services (called here ‘youth clinics’) have been part of the health system for more than 40 years.

In order to explore the challenges and strategies in providing sustainable youth-friendly health-care services, we interviewed 11 health-care professionals working in four different settings in northern Sweden. We analysed these data guided by the five key aspects of youth friendliness, namely accessibility, acceptability, equity, appropriateness and effectiveness.

The informants in this study perceived that professionals working in youth clinics were motivated, interested and knowledgeable about youth, that the clinics ensured confidentiality and prioritized responding to youth health needs, and that they were considered an indisputable part of the Swedish public health-care system. However, they also pointed out some challenges: some youths faced more difficulties in reaching the clinic, the clinic work was not well monitored, the organizational structure and leadership were perceived as unclear and weak, and there were huge inequities in the resources available and the services provided between larger and smaller youth clinics.

## Background

Organized and systematic health services oriented to respond to youths’ needs have a positive impact in their health, through enhancing youths’ trust and access to health care services and, to a certain extent, through promoting health behaviours, e.g. safer sex practices [1–5]. However, research points out that health-care services are, in general, less accessible for young people and few countries have implemented and sustained strategies to make health-care services more responsive to youth health needs [5–8]. The Swedish national network of youth clinics is a good example of health-care services for youths that are integrated within the public health system [9, 10].

Health-care services can provide information on health-related issues, treat youths who are ill, and reach those who are in vulnerable situations [2, 4–6, 11]. However, for health-care services to be beneficial for youths, it is important that they are youth-friendly, or, as defined by the World Health Organization (WHO): accessible, acceptable, equitable, appropriate and effective for different youth subpopulations [5, 12]. A systematic review shows that from young people’s perspective, eight domains are central to a positive experience of care: accessibility of health care, staff attitude, communication, medical competence, guideline-driven care, age-appropriate environments, youth involvement in health care and health outcomes [6]. Youth-friendly health-care services (YFHSs) should be based on supportive policies, respond to youth needs while ensuring confidentiality, promote dialogue with the community, coordinate with other services and implement outreach preventive/promotive activities [4, 12]. Studies exploring health care professionals’ perspectives point out the importance of considering young people as a distinctive group, taking a holistic approach, prioritizing health education and promotion and working in an interdisciplinary way; while barriers such as lack of managerial and financial support, and scarce training and support are mentioned as limiting the implementation and sustainability of YFHSs [13–16].

One important aspect is that of the sustainability of youth-friendly health-care services. In order to be sustained, YFHSs have to be considered part of public health policies, programmes and systems and not organized as isolated initiatives dependent on external support [2, 4, 5, 17]. In fact, the WHO has recently highlighted the need to move forward from YFHSs into youth-responsive health systems [18]. However, progress has been slow, despite the fact that the literature shows that implementing YFHSs is a cost-effective intervention that could contribute to better health among young people [2, 17, 19, 20]. Even in countries where public health systems perform well, health care for young people is usually a neglected area that lacks clear guidelines and political will. Some reasons for that might be: 1) the strong influence of sociocultural norms and policies (e.g. norms and policies regarding age-related competency for informed consent, privacy and confidentiality might hinder the implementation of such services), 2) the biomedical focus of many national health systems does not facilitate responding to youth health needs that are complex and might not be centred in curative services, 3) the limited training on adolescent/youth health issues of health care professionals, 4) funding priorities are given to other age groups and morbidities, 5) effective YFHS need to be linked with other sectors and programme such as sex education in schools [5, 9, 21–23]. Sweden is among the few countries where YFHSs have been integrated within the public health system and sustained for more than 40 years [9, 10, 24, 25], lessons extracted from this experience could be useful for improving health systems’ responsiveness towards young people.

*The Swedish youth clinics: a role model of youth-friendly health-care services?*

Sweden has a well-established network of differentiated YFHSs, called ‘youth clinics’ (Ungdomsmottagningar). According to the national guidelines Swedish youth clinics (YCs) cater for youths aged 12 – 25, although age limits varied between regions, clinics and services [24, 25]. Beginning in the early 70s, a network of youth clinics was developed nationwide in order to improve young people’s access to health information and services. Nowadays it has expanded to over 250 youth clinics across the country [9]. Information is also provided by a web-based national youth clinic (www.umo.se). The clinics are organized into a national association (the Swedish Society for Youth Centres or FSUM) founded in 1988, with the mission of stimulating the development of existing youth clinics [25].

The development and implementation of the youth clinics (YCs) has to be understood in the broader context of the Swedish political, social and cultural norms. In general, the Swedish society has liberal attitudes towards teenage sexual relations, and sexual and reproductive health issues are given priority. Young people have received sex education at schools since the 1950s, abortion has been free on demand since 1975 - all women in Sweden despite their age have the right to have an abortion, although for young women under the age of 18, it is up to the health care provider to judge the maturity of the young person-, and contraceptive counselling and access are easy. In some counties contraceptives are free of charge for young people. Emergency contraceptives are sold over the counter and provided free of charge at youth clinics; cost-free testing for chlamydia and other sexually transmitted infections is easy to access.

However, some backlashes have also been noticed: sex education has been taught less since the nineties and social inequities in youth health have increased [26–28]. Young people from more disadvantaged backgrounds suffer more from health problems, face greater risks to health and have poorer access to health services [29]. Gender and ethnicity also play a role both in health and access to healthcare, i.e. subjective health complains are two times more likely among young women [30], many more of whom report anxiety [31]; Sámi youth — the indigenous populations living in northern Scandinavia and Russia- face specific health challenges such as higher levels of stress connected with experiences of discrimination [32]. Youth from non-Swedish ethnic backgrounds might be at higher risk of being bullied, and refugees have more difficulties accessing healthcare services. In Sweden, as elsewhere, access to health care for youth is modulated by other axis of inequity based on gender, gender identity (trans experiences vs cis experiences), sexual orientation, socioeconomic status, disability, education or ethnicity, i.e. young men, those with trans experiences, those from a non-Swedish ethnic background and those from lower socioeconomic status might face more barriers in accessing healthcare services [33].

Youth clinics can be considered as part of the broader health system, but different from ordinary primary health care and specialized clinics. The Swedish Society for Youth Centres (FSUM) guidelines state that YCs work primarily with health promotion in all areas of youth health with a special focus on sexual and reproductive health. All staff should have a youth-oriented perspective with knowledge of the biological, psychological and social aspects of youths’ development, gender, gender identity and sexuality. It is important that the staff includes professionals educated in andrology, gynecology, psychotherapy and sexology and the goal is to be able to help all young people with their questions, whatever they may be. Youth clinics do not deal with severe or complex somatic or psychiatric problems or diseases. However, since young people might access YCs more easily than other health care services, YCs might become an entry point for youths into the health system, connecting them to primary health care or specialist care for assessment and treatment [34, 35]. The clinics are mainly run by county councils, e.g. within the ‘first line’ primary care services, although municipalities and private organizations run some of them or may collaborate in certain clinics. How each clinic is staffed and which services are provided vary. According to the (FSUM) guidelines, the minimum staff of a YC includes a midwife and a social counsellor or psychologist, with a physician working part-time. In order to improve accessibility, most youth clinics are located off the premises of general health services, and consultations are free of charge. In addition, promotion work, mainly through school visits of 15-year-old pupils to the clinics, increases young people’s awareness of the services offered by youth clinics [10].

There is concordance between the Swedish Youth Clinics’ handbook, published in 2015, and the WHO criteria for youth friendliness (Table 1). The FSUM’s handbook serves as a national guide (even if not legally binding) for youth clinics [34].

**Table 1:** World Health Organization (WHO) youth friendliness criteria (modified from [4]) and related aspects contained in the Swedish youth clinics’ most recent handbook [34] (table developed by the authors)

WHO domain	Strategies within the FSUM handbook/policy
Accessibility: • Policies and procedures to ensure that services are free or affordable • Convenient opening hours and location • Youths know about the services and how to get them • Community supports services and understands benefits • Outreach work towards community	• All visits should be free of charge • Staffing, opening hours, facilities and methods adapted to local setting • Youths should be able to make contact through telephone or IT-based solutions • Promote drop-in visits • Outreach work through school class visits to the clinic, visits to schools and participation in arrangements in the community
Acceptability • Policies and procedures to ensure confidentiality • Attitudes of providers: provide information, support youths’ decision-making, motivated, non-judgmental, able to dedicate time • Adequate environment: privacy, physical safety • Youths monitoring the services	• Staff must meet youths with respect and understanding in a safe environment • Youths who seek help should understand the rules regulating confidentiality and privacy • Dedicate time to quality and safety work • Evaluate the services provided through surveys for youths
Equitable • Policies and procedures that do not hinder services and that consider aspects that might be an obstacle for equitable care • Professionals treat all youths with equal respect, independently of their status.	• Provide equitable care regardless of personal characteristics, place of residence, age, gender, disability, education, social status, ethnic and/or religious affiliation or sexual orientation • The youth clinic must have good procedures for interpretation in different languages • Usage of a norm-critical approach
Appropriateness • Care to fulfil needs of youths is provided through health services and/or referral to other services • Professionals respond adequately to youth health needs and take other issues that can affect the youths into consideration	• To have a holistic approach, see youths in the social context where they are embedded • Youth clinics should always work on behalf of the youths • Multidisciplinary teams with broad expertise to meet the needs of young people • The psychosocial perspective is integrated in all visits
Effective • Professionals with required competence • Care is guided by protocols and guidelines • Equipment and other resources for adequate care exist	• National guidelines and rules should be applied, for example concerning partner tracing and treatment of sexually transmitted infections • Economic conditions that support activities in relation to the goal • To have a common understanding of what quality work constitutes of
• All domains in general	• All the work should be based upon human rights and the youth clinics should be part of the democratic society • Work for promotion of youths’ health • Youth clinics have a mission to promote sexual and reproductive health in a public health perspective

Despite the fact that Swedish youth clinics constitute one of the most comprehensive and consolidated examples of a nationwide network of health-care services for young people, to the authors’ knowledge, there are no studies exploring how they work to operationalize the criteria of youth friendliness and how and why the YCs have become sustainably integrated with the health system. Some possible factors might be related with Swedish progressive policies in relation to youths’ sexual and reproductive health and rights and autonomy, public investment on health, education and social services, and the harmonizing and coordinating role of the Swedish Association of Youth Clinics (FSUM) which provides continuing professional development and training, produces highly respected guidelines, policies and best practices, and ensures exchange of information among YCs’ members. The exploration of the Swedish case could shed some light on: 1) what the main challenges in providing and sustaining youth-friendly health-care services as part of the health system are, and 2) what strategies can be used to overcome them.

This study consequently aims to explore the challenges and strategies involved in providing sustainable youth-friendly health-care services, from the perspective of professionals working in youth clinics in northern Sweden.

## Methods

### The setting and informants

The study was conducted in a county in northern Sweden. It is part of a larger study assessing the youth-friendliness of YCs in northern Sweden [36]; this specific county was chosen because it was similar to other northern counties and had a variety of YCs -in terms of size, focus, location-, that allowed to explore diverse experiences. The county has a population of around 200,000, with 11% being aged 18–24. There are four youth clinics in the county, located in the largest cities and towns. The first one started functioning in 1984. In some of the municipalities where there is no youth clinic, midwives or other professionals might dedicate specific hours or days to attending to young people. Youth clinics in this county are mainly funded and managed by the county council, as with health centres and hospitals, although collaboration with the municipalities also takes place, i.e. certain professionals are contracted by the municipality.

Eleven professionals with varying university education and positions were interviewed: three midwives, three social workers/counsellors, two nurses, one psychologist, one family doctor and one administrator. Four of them had managerial positions. The ages of the informants ranged from 39 to 62. They had been working within youth clinics for between six months and 18 years, and all but one had been working in the youth clinic where the interview was conducted for more than four years. For this study we conducted 11 semi-structured individual interviews. Ten interviews were conducted with professionals working in three out of the four youth clinics existing in the county, while one interview was conducted with a health-care professional offering differentiated hours for youth within a primary health-care centre in a rural area. We included it because we considered that this setting could contribute with very useful information about the difficulties and strength of offering services with a similar approach to a YC, but much limited resources —time, professionals-, in rural areas. Two clinics were located in large cities, offering services Monday to Friday, and were located in a central area separated from other health-care facilities. The third clinic offered services once a week and was located within a primary health-care centre.

### Data collection

In order to contact the potential informants for this study, we first approached the head of each youth clinic and informed about the profile of participants from the clinic that we were interested in interviewing. The head of each youth clinic then made contact with different professionals to ask for their participation. The interviews were then scheduled through email contact, and questions from the informants regarding the interview were further clarified through email and telephone contact. The semi-structured interview guide was organized in a thematic manner, inspired by the WHO domains of youth friendliness with a focus on challenges and strategies [4]. Relevant issues that emerged within the first interview and were not part of the interview guide were further explored in the subsequent interviews (with other informants), following an emergent design.

Data collection was carried out from April to December 2015 by two of the authors (ST and DM). The interviews were conducted face-to-face in the YCs. The interviews were digitally recorded and lasted on average 53 min. They were carried out in Swedish, which was the mother tongue of both the interviewers and the informants.

### Data analysis

Interviews were transcribed verbatim, and analysed using thematic analysis in relation to the WHO domains of youth friendliness [4, 37]. Each interview was read systematically and then the transcriptions were coded. The data were coded manually using the original Swedish transcripts section by section, and codes were afterwards translated from Swedish into English. First, the sections of the interviews that referred to each of the five domains were identified and marked with different colours. Second, each of those sections was coded. Three of the authors (ST, DM and IG) participated in the coding process. During these first two steps, issues that we considered did not fit within the five domains, but referred to challenges and strategies in ensuring youth friendliness, were also marked with different colours and coded. Third, a list of all the emerging codes was developed and all the codes that were related to each other were grouped in subthemes. Finally, through getting back to the text, connections between the subthemes were identified and themes emerged. Preliminary themes were first negotiated between ST, DM and IG and later on with all the co-authors.

### Ethical considerations

This study was approved by the Regional Ethical Board (Dnr. 2015-190-31Ö), which works under the Government of Sweden. Verbal and written informed consent were sought from each informant, after assuring them that participation was voluntary and that they could cancel their participation at any moment. Any information that could expose the identity of the informants was removed from the transcripts. Confidentiality was ensured during the reporting, so that the identities of the informants were secured.

## Results

Four themes emerged from the analysis of the data. The theme ‘Meeting youths on their own terms— the key to ensuring a holistic and youth-centred care’ was related to the acceptability and appropriateness of the services. The theme ‘Organizational challenges and strategies in keeping professionals’ expertise on youth updated’ referred to the domain of effectiveness. The theme ‘Youth clinics are accessible for those who know and can reach them’ was related to the domains of accessibility and equity. The theme ‘The challenge of combining strong directions and flexibility in diverse local realities’ touched upon different aspects of the WHO domains but at another level; it focused on the struggle to sustain the youth clinics organization and their goals within the broader health system. A summary of themes, subthemes, strategies and challenges can be found in Additional file 1.

### Meeting youths on their own terms — the key to ensuring a holistic and youth — centred care

Informants considered that YCs made great efforts to ensure that they were youth-centred, meaning that youths’ needs and opinions became in focus and prioritized. Youth-centredness was described as meeting youth where they were, treating them with empathy and in a respectful manner.

Professionals considered that providers’ attitudes and approach to youth was central to achieving youth-friendly services. Professionals considered that they should approach youths as equals, avoid adult-centric, patronizing attitudes and be aware of how their own prejudices about youths could affect their communication with youth people. They recognized a number of features that professionals working in YCs should incorporate in order to ensure that services were offered in a youth-centred way, such as being open-minded — not getting shocked with youths’ questions and requests and not imposing their own moral views —, interested in youth, respectful, welcoming, non-judgmental, not patronizing, flexible and curious, and enjoying their work.

*I have said, the day a youth enters my door at the office and I’m not curious, then I quit. You need to feel interest in how it is for them.* (Informant 1, Setting 1).

Informants stated that a youth-friendly clinic needs to have a holistic approach to health, meaning that youth should perceive that they can come to the clinic for information or with questions or issues about relationships, emotions, etc. Informants considered that ensuring that youth clinics were perceived as different from other health-care services was important, since young people must feel that they can come to the YC to consult on things not directly related to specific physical or mental problems.

*Here it is possible that they can come with an open question and then we help them place it in the right box. In an ordinary health-care centre there is no space to just come there because you have a simple question or because you have a question but there is actually something else behind.* (Informant 5, Setting 3).

Being located away from other health-care facilities was considered an effective way to be perceived as ‘not too much about medical care’. It was considered beneficial in regard to distancing youth clinics from ‘ordinary’ health-care services as well as to preserving the autonomy of the clinics. However, this was not always possible to do, especially in smaller youth clinics located within existing health-care centres.

*The risk is that it becomes a little bit too much ‘health care’ when we are situated within a health-care centre. Of course we are affected by the ones we have around us, the nurses, the doctors and everyone working here.* (Informant 1, Setting 1).

One aspect that informants considered especially important was ensuring confidentiality and assuring youths about how seriously this was taken in the clinics. Informants considered it key to inform the young people about confidentiality and what exceptions could apply in order to reassure youths of their rights. The space of the clinics should warrant spatial confidentiality in terms of, for example, rooms being well isolated and soundproof, keeping doors closed and avoiding knocking at doors during consultations. The challenge of preserving the spatial confidentiality of the clinic while at the same time projecting an image of openness and non-medicalized services was mentioned.

*We want it to be open but at the same time … I don’t know, it’s double. Red lights, for example, are common. The ones you turn on that can be seen outside the door. This is something we have discussed. But there are pros and cons because at the same time we don’t want it to be like in a hospital. Now, a closed door means you should not enter. It is only in the case of an emergency you may knock on the door.* (Informant 2, Setting 2).

Informants were aware of the different challenges to confidentiality depending on the location of the clinic. In small places where everybody knows each other confidentiality and privacy might be harder to ensure. Central locations outside health-care services might seem more private, but once the location becomes well known it might hinder access for certain youths that might fear being seen going into the clinic.

*The youth clinic is located across from a cafe… and that has made it difficult for some youths… to dare to enter. Because they are seen by adults…* (Informant 3, Setting 2).

In order to offer good services, informants considered it important to establish dialogue with young people, and to be attentive to youth opinions and suggestions about the services. Informants mentioned the importance of asking the youths, taking on board their opinions, listening to what youths want and the changes they suggest. They described informal ways to do this. One of the clinics had a guestbook in the waiting room and also provided notepaper for the youths to write and put in a box anonymously. Some of the clinics have also developed more formal strategies to get feedback from young people, such as using a youth survey conducted annually. Based on the suggestions of young people, one of the YCs started opening during some evenings, although this activity was lately discontinued. Youth opinions were considered the ultimate quality assurance system, as one informant expressed:

*What we do and what I think is good is that we have a mailbox in the waiting room and notes where the young people visiting us can give suggestions […] And that is something to embrace much more than if you might get to hear it, yes, if the boss sits and goes through the guidelines. So I embrace it more if it is written on a note from someone who has been visiting.* (Informant 4, Setting 2).

### Organizational challenges and strategies in keeping professionals’ empirical expertise on youth up to date

Informants considered that professionals working at youth clinics were knowledgeable about youth health. They also described how the choice of who was going to work in a youth clinic was based on the individual professional’s motivation and interest in youth health, which ensured continuous improvement of competence and skills. Such motivation and interest in youth needs could not be replaced by any amount of training.

*You can take every course you want, but if you do not feel comfortable at work or if you feel you are in the wrong place or if you feel you are not in a position to do quality work, then I think no course helps.* (Informant 4, Setting 2).

Learning to respond to youth needs was described as a never-ending process, since youths’ needs change over time. Contact with youths both forced professionals to be updated and constituted a great opportunity to keep themselves up to date.

*It is the youth that teaches me, you could say; they update me, so to speak, because it is clear, I do not live in their world, in the same way as they do. But they keep me updated.* (Informant 3, Setting 2).

Teamwork was considered important both as a way to ensure a comprehensive response to the multiple and interconnected youth health needs and as a means to ensure that professionals feel supported and challenged, by being exposed to diverse perspectives.

*When I feel I cannot handle it properly, I make contact with someone who does. So I do not let youths down when they need me most.* (Informant 7, Setting 2).

Continuous refreshing and training was not equitably available to all youth clinics and was less easy to ensure in settings with fewer professionals. For example, while the team in a larger youth clinic was enrolled in periodic training sessions to get a Lesbian-Gay-Bisexual-Trans-Intersex-Queer (LGBTIQ) certification, this was not the case for professionals attending to youth in a municipality with no YC. Motivation without continuous training and support was not seen as enough to offer good services.

*It feels like this is work expected to be run by the left hand… It’s expected to proceed smoothly and work out but I never get to participate in any training courses.* (Informant 6, Setting 4).

Professionals identified the scarcity of appropriate continuous monitoring and evaluation processes as a major challenge. They considered that youths’ opinions on the services would be relevant and legitimate as a way to monitor the YCs. In addition, evaluation routines differed between youth clinics and were dependent on individual initiatives. Heavy workload was also mentioned as hindering the opportunity for systematic reflection and improvement:

*To go through how the year has been, what we have done, things that have been bad, what to improve, that is very important. But we haven’t had time, we have lost it in between everything else […]. We need to refresh the framework, aim and purpose […]. For my part there has been such hard pressure with work so I have just been going with the flow.* (Informant 1, Setting 1).

### Youth clinics are accessible for those who know and can reach them

Informants considered it important to make sure that it is easy for youth to reach the YC and pointed out that YCs implemented a number of strategies to ensure accessibility. The fact that consultations were free of charge was considered to be key, as well as the fast and diverse pathways through which young people can get in contact with YCs, i.e. drop-ins, phone consultations, booking an appointment, or consulting the web-based clinic at umo.se. The opening hours of the YCs were considered to be a key factor in shaping accessibility: longer opening hours facilitated young people’s access, while clinics that were open only once a week limited access.

*…if is it open every weekday, a young person in need might feel a little better one day and take courage and seek help, but if it is open one day a week it is certain that this person will have just that courage that day.* (Informant 7, Setting 2).

Informants reflected on how the working hours of the clinic could be improved to better fit the hours when young people might be freer to come, or when they might find it easier to come without having to tell their parents, teachers or employers, such as earlier in the morning, later in the evening or during the weekends. However, having opening hours that might be friendlier for young people conflicted with staff preferences. In smaller youth clinics with fewer staff, this was particularly problematic.

*It will cost more money to keep it open during evening time and that is hard to get. We are not allowed to work alone and that is also one thing, considering I always work alone. To have me working here alone in the evening would not be good from a safety perspective. So both finances and logistics put a spoke in the wheel. (*Informant 6, Setting 4).

Preventive and promotional activities in coordination with other sectors were considered important. In the largest clinics, some professionals were engaged in a number of community-based activities targeting different youth subgroups, i.e. work with schools, workshops with unaccompanied young refugees and unemployed youth. The smaller the clinic, the harder it was to maintain good and continuous services in the clinic and engagement in community-based activities.

*We are in need of more time to meet other vulnerable youth groups. I want to participate in community-based work; it is very, very important… Schools, social services, police…, these are important authorities, and it is important to cooperate with them. The problem is that to cooperate I have to join the meetings.* (Informant 7, setting 3).

Despite this conflict between community-based and clinical work, youth clinics have managed to sustain good collaboration with schools. School pupils’ visits to the YCs were part of the routine activities of the YCs and were seen as a strategy to promote the youth clinic among their potential users.

*Lots of youths who come here for the first time, they remember that sometime, maybe a couple of years ago, they came here with their school. Many say so.* (Informant 2, Setting 2).

Professionals were satisfied with the services provided but critical about some points. They recognized that access to the clinic was not equitable for all youths and mentioned a number of intersectional dimensions that might hinder it: youth with disabilities, youth from non-Swedish ethnic backgrounds and young men accessed the clinics less often.

*The tradition is that it is a girl clinic and it becomes something like it is just girls who have problems. Or should be responsible for protecting themselves and so on…* (Informant 8, Setting 2).

Informants mentioned that the majority of the staff in the YCs are women and that having more men on the staff might attract more young men to come. Informants also mentioned that those youths who might be in most need (i.e. those with severe psychosocial problems, unaccompanied refugees and youths with disabilities) might not have enough strength to reach the youth clinics, so other ways of reaching them might be needed.

*Unaccompanied young refugees… They do not have the opportunity to get to the youth clinic. We are doing outreach work with them, visiting the schools where they are… That makes it easier to get in contact with them… Otherwise it’s hard…, there are different languages … We do it because we have extra resources for that but also… I think it’s a matter of fairness.* (Informant 4, Setting 2).

In addition, despite acknowledging that sexuality, sexual identity and orientation accounted for an important number of the consultations at youth clinics, informants thought that LGBTIQ youth might find it harder to access youth clinics, especially in smaller places.

### The challenge of combining strong directions and flexibility in diverse local realities

Trying to respond to youth health needs in diverse contexts and with different amounts of resources and political commitment meant that YCs could look very different in terms of size, opening hours and staff, but also in terms of age limits and prioritization of health needs and topics. This influenced the services they offered and the way they worked. Resource constraints and context characteristics determined the types of services available, while professionals working in each clinic struggled to make the best of their situation.

In small youth clinics it was impossible to work as a team, a fact that was believed to be hindering quality as well as putting too much burden on the existing professional(s). But too large teams might also make it more difficult to keep a ‘joint spirit and mission’, especially when there is too much turnover of staff.

*We don’t want to become huge because we… The more there are of us at one clinic, the harder it is to keep a line.* (Informant 5, Setting 3).

Informants perceived differences partly as unequitable and unfair for young people served by smaller and less comprehensive clinics. At the same time, they thought that, to some extent, differences were not undesirable but a sign that youth clinics were flexible and adapted services and approaches to the specific needs of the youth they served.

*The conditions vary a lot between different towns and places so it is hard to find a solution that will be good in all places. Because of this we might have to allow inclusion of some flexibility […]* (Informant 5, Setting 3).

On the one hand, autonomy was considered positive for the YCs. On the other hand, it was also seen as a sign of the lack of strong directives from health authorities and the communication gap between higher-level managers and professionals at the clinic.

*It is just that with leadership. It is like one joint is missing. Decisions have been made that one wants a youth clinic; the politicians, they want that very much. But something is missed in between them and us.* (Informant 10, Setting 2).

Diversity also made it more difficult to coordinate among the different professionals within the YCs, i.e. different age limits for mental health than for sexual health made it difficult to internally refer certain youth, between youth clinics, and with other services — i.e. health-care centres might be uncertain about the services provided in a specific YC and/or about the age limits, since they vary from one location to another and even within the same clinic depending on the professional consulted.

YCs might be diverse and have weak management structures within the health-care system, but this did not mean that they lacked a strong vision and mandate, quite the opposite. Rather, informants considered that the FSUM policies, guidelines and handbook formed the backbone of the youth clinics mission. They endorsed them as an inspiring code of conduct that they were affiliated to, or as one informant expressed: “*there is no one up there somewhere* [who created the policies and handbook]*, but it is us*” (Informant 2, Setting 2). In that sense it was more a declaration of principles than a technical guide, a mission and vision that allowed YCs to be unified by a core goal while at the same time giving each of them room to operationalize it in diverse ways.

*You need to have the concept of youth clinic; it is something special. It is not an ordinary primary care centre; I think that this is important. If you read the FSUM’s declaration, that’s something else than the primary care centre. That’s how it is.* (Informant 1, Setting 1).

## Discussion

Professionals in this study felt that Swedish youth clinics adhere to the WHO domains of youth friendliness, both at the level of what was proclaimed in policies and guidelines and their implementation. However, challenges were also pointed out, especially in terms of ensuring equity, quality and organizational support.

In line with other studies, this paper emphasizes the importance of professionals’ attitudes in ensuring the acceptability and appropriateness of services [6, 8, 11, 15, 38, 39]. A strategy used to ensure professionals’ motivation was to make efforts in the recruitment process to select professionals not only with the proper skills and education but also who were highly committed to working with youth. However, in smaller rural clinics the recruitment of health-care professionals is more difficult and candidates to choose from are not usually available [40, 41]. While high personal commitment might be key, it needs to be supported by an organizational structure that provides continuous training and that enables the time and freedom to put it into practice [15, 39, 42]. As stated in the results, ‘Working with the left hand’ does not ensure that the services provided are effective and might contribute to frustration and demotivation. There have been efforts to refresh training of professionals working in YCs in northern Sweden, especially in regards to LGBTIQ certification. However, as this study points out, inequities between clinics in access to such programs exist and affect the quality of the services provided. Beyond continuous training of already employed professionals, incorporating training on youth health during undergraduate and postgraduate studies adds to the sustainability of these services, and constructs youth health as (also) the responsibility of the diverse health care professions. However, this is a strategy that has been scarcely implemented worldwide.

Ensuring confidentiality has been highlighted in other studies as something that youth take into great consideration when deciding to seek care [6, 8, 9, 15, 38]. An important point raised in this study was the balance between ensuring that the YCs ensure confidentiality and privacy for their users, while at the same time remaining visible and presenting an image of openness. As other studies have also pointed out, confidentiality is more difficult to ensure in smaller places [40, 43, 44]. Informants in this study were aware of this problem, although they did not come up with potential strategies to handle it.

Several reports and studies from other contexts such as Australia and Vanuatu have stressed the importance of overcoming structural or operational barriers, such as; inconvenient hours of operation, high cost of service, to increase accessibility [45, 46]. Our study suggested that the structural barriers were tackled by three important strategies implemented to enhance accessibility mentioned in this study 1) ensuring that services were free of charge and 2) ensuring that opening hours were generous and 3) ensuring that youths had diverse ways of getting to the clinic. An Australian study found that youths considered important to be able to reach the clinic without the requisite of a booked appointment [46], a strategy that was also valid for the Swedish youth clinics system of ‘drop in’. The concept of ‘drop-in’ consultations was considered especially valuable and the main form of access in smaller places with fewer working hours. However, this study also stresses that ensuring that opening hours are youth-centred is not always easy to implement, and leadership support and resources are needed to implement such changes. Other studies pointed out that opening hours and appointment arrangements are key to ensure accessibility [5, 6, 11].

In this study, youth centredness relied on staff’s respectful attitudes and confidentiality, and also on ensuring that YCs had a holistic approach to health. Swedish YCs have managed to deliver the message that they are different from other health-care services and that youth are welcome to go to their YC with any issue or problem. This approach makes YCs more appropriate for responding to the complex needs of young people, which often are not limited to one specific health problem, or when the reason for consultation might be merely the starting point for revealing other issues. Previous studies have also highlighted the importance of ensuring that youth and their needs are in focus in order to ensure that health-care services are appropriate [4, 47]. In that sense, youth-centred care is not different from person-centred care, and YCs might be in the vanguard of how other health-care services might approach health and users in a more holistic way and with better user satisfaction [48, 49].

Purposively locating the YCs outside health-care facilities helps to make them more accessible for young people. This strategy did not seem feasible in smaller places and funding issues were claimed to be the reason for that. The WHO guidelines point out that efforts should focus on making existing health-care services youth-friendly instead of developing new service delivery points to youths; however, the guidelines also stress that differentiated services might be more accessible, especially for marginalized and stigmatized groups of youths [4]. Evaluations comparing the cost-effectiveness of the two approaches are lacking.

One possible disadvantage of this image of YCs being ‘not so much about medical problems’ might be that youth clinics might be losing an opportunity to be the entry point into the health system for severely ill young people who might refrain from accessing any health care service (e.g. psychiatric care). Although the role of YCs is not to treat those youths who are severely ill, they can play an important role in detecting youth at an early stage and directing them toward adequate support. The debate on which should be the role and level of competence of YCs in terms of health issues beyond sexual and reproductive health, and the connection between YCs and other health care services is ongoing and very relevant in order to facilitate youths “navigation” within the different health care services in Sweden. The literature has already pointed out that youths get less time for consultation in ordinary health services, and risk becoming an ‘invisible’ group [50, 51], and some Swedish youth risk ‘falling between’ services like YC and specialist care, or between somatic and psychiatric care, or child and adult services [52].

The YCs in Sweden may be looked upon as a flagship within health care, as they provide young people with access to information, counselling and support related to sexual health issues such as birth control, pregnancy and STI testing, and can equip young people with the attitudes, knowledge and skills they need to make informed choices now and in the future, enhance their independence and self-esteem, and help them to experience their sexuality and relationships as positive and pleasurable [53].

Two issues that this study identified as big challenges were related to the difficulties in institutionalizing community work to enhance accessibility and the scarcity of specific strategies to ensure equitable access to YCs for all youths. Community orientation is commonly recognized as one of the most challenging attributes to achieve when implementing a primary health-care approach within health systems [54]. In terms of YCs, despite their holistic and youth-centred approach, outreach and community work was hard to make compatible with office consultations, was less prioritized and harder to sustain among the smallest YCs. A noteworthy exception is the coordinated work with schools that all YCs do. YCs have used the strategy of school visits for many years and it seems that it works for promoting the existence and services of YCs among their potential users. This strategy seems easy to transfer to other countries and is not demanding in terms of the resources involved.

Despite the long-term work of YCs in this part of the country, informants were well aware of the existence of inequities in access for youths, i.e. based on gender, gender identity or ethnic background. While large clinics were coordinating activities with other organizations to reach youths that might be in vulnerable situations, e.g. newly arrived refugees, this was not possible in smaller clinics. The fact that YCs are mainly used by young women was mentioned, and it also aligns with the statistics from the YCs in this county where between 75 and 90% of the people consulting are women. Interestingly in the largest YC that offered mental health services, the percentage of young men accessing such services rose to 29% (while they accounted only for 17% of the total consultations). This can also be related with the fact that the majority of the staff in the clinics are also women. The fact that YCs are mainly used by young women have also been reported both in Sweden and in YFHSs in other settings [9, 55]. The other axes of inequity mentioned in this study — gender identity, sexual orientation, ethnic background and disability — deserve further exploration. Other studies have pointed out that the domain of equity is the one that is usually less developed when YFHSs are implemented [2, 42, 56].

The most recent WHO recommendations push for moving beyond youth-friendly health-care services towards youth-responsive health systems. This study supports this approach and unveils some of the challenges and strategies involved in ensuring that YFHSs are fully integrated and sustained within the health system. It also underscores that in order to be responsive to youth people’s needs, health systems should be people-centred, namely: 1) place people’s voices and needs first, 2) be guided by principles of quality, safety, longitudinality, closeness to community and responsiveness to changing requirement, 3) acknowledge the centrality of human interactions in service delivery, and 4) be grounded on values of rights, respect and equality [57].

According to the informants, in the Swedish context the existence of youth clinics is not questioned. More than 40 years of work and the existence of a national organization make it possible for YCs to be sustained and consolidated as a legitimate part of the public health system. This is not the case in many other countries where resource constraints and the controversies surrounding YFHSs might hinder the likelihood that policies and services for youth get prioritized. This finding underlines the importance of political and socio-cultural aspects in ensuring that health systems are responsive to youth [15, 42, 58].

The way the YCs started (small scale) and the way they have continued to work — in a flexible, bottom-up fashion, where a national organization integrated by the YCs themselves serves as a guiding structure — has been successful in ensuring that a certain ‘style’ of services has become the rule. Swedish YCs have been able to build a style of working that differentiates them from ‘ordinary health-care services’ and that makes them realize most of the domains of youth friendliness. This flexibility has allowed them to adapt their services to local realities and to have freedom in the way they work. This way of implementing YFHSs in pilot sites has been proven to be useful in other settings [15, 38], but this is the first time that a study has pointed out this flexibility and freedom as a feature for sustaining YFHSs within a national health system structure. One key aspect that brings sustainability to this bottom-up approach might be the role of the FSUM in building a structure that brings coherence and unity beyond the specific characteristics of each region and clinic. Without such a guiding role, local flexibility and freedom could have led to complete anarchy and losing the mission and vision of the YCs.

However, while the FSUM has a legitimate position in guiding the YCs, and makes big efforts to influence policymakers, it has no decision-making power within the health system structure. Thus, since YCs depend upon different managerial structures (some upon municipalities, most upon county councils, a few upon private institutions), it is unclear who should be responsible for ensuring that YCs do what they are supposed to do. That lack of clarity and leadership was mentioned as a key challenge to sustainability in this study. Informants reflected on existing inequities between large and small YCs, and the way resources were allocated was not transparent for professionals working in the YCs. Despite YCs being well legitimated within the Swedish health system, they seem to face the same problems in terms of resource constraints and increased workload as the other primary care services [59]. The funding for youth health recently allocated by the government might improve this situation.

### Study limitations

This study has limitations. The translation of the codes into English might have led to losing part of the meaning in the translation. This study is based in northern Sweden, where the context may differ from other settings in the same country, i.e. lower population density, longer distances to health-care services, and smaller towns. The findings have to be interpreted with this context in mind. However, the fact that we included clinics of different sizes and locations, and the fact that the core guiding values and aims of Swedish youth clinics are shared nationally through the FSUM, might add to the transferability of these findings. The way informants were approached (through the head of the clinic) might have led to not including the more critical voices. The study only captures the providers’ side; in the future it would be interesting to contrast professionals’ views with those of the young users of the clinics.

## Conclusions

To the best of the authors’ knowledge, this is the first study to explore the challenges and strategies involved in implementing YFHSs within the Swedish youth clinics. The long history of the youth clinics and their well-established national network make them a good case for further study on this issue.

Swedish youth clinics’ policies adhere to the WHO domains of youth friendliness. The health-care professionals interviewed in this study stated that YCs’ staff are motivated, interested and knowledgeable about youth and that the clinics ensure confidentiality and a youth-centred and holistic approach. However, challenges were also pointed out, especially in terms of ensuring equitable access to different youth subpopulations, improving monitoring routines, and ensuring training and competence for all professionals, independently of the location and characteristics of the clinic. Youth clinics were perceived as an indisputable part of the Swedish health system, but organizational challenges remained, and these were pointed out in terms of weak clear directives and leadership, heavy workload, local/regional diversity and unequitable distribution of resources.

## Additional file

Additional file 1: Themes and subthemes, with a summary of the challenges and strategies identified. (DOCX 16 kb)
