# Supplementary material for: Challenges and strategies for sustaining youth-friendly health services — a qualitative study from the perspective of professionals at youth clinics in northern Sweden
Source: Reprod Health. 2016 Dec 21;13:147. doi: 10.1186/s12978-016-0261-6 (PMC5178097; doi:10.1186/s12978-016-0261-6)
Supplement: Additional file 1: — Themes and subthemes, with a summary of the challenges and strategies identified. (DOCX 16 kb) [file 12978_2016_261_MOESM1_ESM.docx]

Supplementary material 1

Themes and subthemes, with a summary of the challenges and strategies identified

|  | **Meeting youths on their own terms – the key to ensuring a holistic and youth-centred care** | | |
| --- | --- | --- | --- |
|  | **Subthemes** | **Challenges** | **Strategies** |
|  | Professionals’ interest, welcoming attitudes and motivation towards youths |  | Make sure that professionals display appropriate attitudes: welcoming, interested, non-judgmental |
|  | Ensuring ‘spatial’ and informed confidentiality | Hard to have spaces that ‘look’ open and are well known and welcoming while at the same time ensuring privacy for young people coming  In smaller places privacy is harder to ensure | Make explicit to young users how confidentiality is warranted |
|  | The importance of being attentive towards youths’ opinions | Mechanisms to collect youths’ opinions depend on each YC interest and capability | Gather, value and act upon youths’ opinions |
|  | Youth clinics should not be too much about medical problems | In smaller places YCs are within health-care facilities, harder to be seen as ‘not too clinical’ | Make explicit that the focus is youth and their concerns  Be located outside health-care facilities |

| **Organizational challenges and strategies in keeping professionals’ empirical expertise on youth up to date** | | |
| --- | --- | --- |
| **Subthemes** | **Challenges** | **Strategies** |
| Youth clinic staff are experts on youth | Smaller YCs with few staff find it harder to keep updated  Smaller YCs lack multidisciplinary teams  In large YCs it’s harder to keep a shared vision | Recruit professionals who are motivated and interested  Existing training programmes, i.e. LGBTIQ certification, FSUM conferences  Contact with youths makes them feel the need to be updated  Work in multidisciplinary teams |
| Improving heterogeneous evaluation routines | Monitoring routines not institutionalized  Collected indicators not perceived as the most appropriate  Workload disengages monitoring and critical quality assessment processes | YCs implement strategies for monitoring quality depending on time and resources |

| **Youth clinics are accessible for those who know and can reach them** | | |
| --- | --- | --- |
| **Subthemes** | **Challenges** | **Strategies** |
| The importance of offering free services |  | Free consultations |
| Fast and diverse pathways to get access | Hard to widen opening hours to better suit youth perceived preferences  In smaller YCs shorter opening hours | Combine diverse ways to get to the YC: booked appointments, drop-in, Internet |
| Wanting to improve strategies for outreach work | Smaller YCs cannot engage in other outreach work due to staff shortage | School visits to the clinic  Larger YCs can do outreach activities with other sectors |
| Not all youths have equal access | Certain groups perceived as accessing less YCs: young men, LGBTIQ youth, youth with disabilities, from non-Swedish ethnic background, young people with more severe health problems |  |

| **The challenge of combining strong directions and flexibility in diverse local realities** | | |
| --- | --- | --- |
| **Subthemes** | **Challenges** | **Strategies** |
| Trying ways to respond to youth health needs with various resources | Harder to sustain ‘comprehensive’ YCs in smaller places  Unclear how much YCs should get involved in mental health issues | The existence of YCs is not disputed  The focus on SRH is well sustained  The model of YCs is flexible, so that it can be ‘adapted’ to different contexts and resources available  Each YC decides upon certain working issues, which allows for better adapting to local needs |
| Leadership from above the clinic is the one joint missing | YCs have unclear place in the regional and national health system structure  No clear managerial guidance beyond the clinic |  |
| The participatory guiding role of FSUM | FSUM has no ‘legal’ power to ensure that guidelines are followed | YCs organized in a national organization: FSUM  FSUM links together the YCs at a national level and provides support  Guidelines and policies coming from FSUM embraced by YCs as their own legitimacy |
